# In Vitro Fermentability of Soybean Oligosaccharides from Wastewater of Tofu Production

**DOI:** 10.3390/polym14091704

**Published:** 2022-04-22

**Authors:** Yuling Wang, Chunrong Li, Zhengxin Shan, Sijia Yin, Yue Wang, Congcong Wang, Tianhui Liu, Nifei Wang, Qingbin Guo

**Affiliations:** 1 School of Life Science and Technology, Henan Institute of Science and Technology, Xinxiang 453003, China; wangyuling634@163.com; 2 State Key Laboratory of Food Nutrition and Safety, College of Food Science and Technology, Tianjin University of Science and Technology, Tianjin 300457, China; 18847962lcr@mail.tust.edu.cn (C.L.); shanxzz789@163.com (Z.S.); 19846019@mail.tust.edu.cn (S.Y.); wy18317582018@163.com (Y.W.); 3 Tianjin Shanhaiguan Bean Products Co., Ltd., Tianjin 300000, China; wang_cong_cong@126.com (C.W.); m1572330210@163.com (T.L.); 4 College of Biotechnology, Tianjin University of Science and Technology, Tianjin 300457, China

**Keywords:** soybean, oligosaccharides, in vitro fermentation, short-chain fatty acids, gut microbiota

## Abstract

Soybean oligosaccharides (SBOS) isolated from wastewater of tofu production were studied in terms of their structural characteristics and in vitro fermentation by human fecal inocula. Three sub-fractions named Z1 (14%), Z2 (13%), and Z3 (17%) were obtained by Sephadex G-15 column separation. Z1 contained mainly stachyose; Z2 and Z3 contained stachyose, raffinose, and sucrose with different relative percentages. The in vitro batch fermentation model of human intestinal bacteria including 0, 12, 24, and 48 h was used to investigate the fermentation characteristics of SBOS. According to the results, during the fermentation process, the molecular weight of oligosaccharides decreased significantly with increasing fermentation time, indicating that oligosaccharides could be utilized and degraded by the colonic microbiota. Furthermore, SBOS could significantly promote the production of short-chain fatty acids (SCFAs), especially acetic, propionic, and butyric acids. SBOS increased the abundance of *Firmicutes*, while that of *Proteobacteria* was decreased. Additionally, SBOS could promote the proliferation of *Dialister*, *Bacteroides*, and *Akkermansia* at the genus level. Therefore, SBOS can be potentially used as prebiotic promoting gut health.

## 1. Introduction

Tofu, also known as bean curd, is a food product prepared by coagulating soy milk and pressing the resulting curds into solid white blocks of varying softness [1]. In tofu production, by-products such as soy whey, bean dregs (almost 15 million tons each year), and wastewater are produced [2,3]. These by-products are mostly used as animal feeds, fertilizer, and in biofuel production, or even left for waste, causing environmental issues such as bad odors and pollution to surface and groundwater [1,4]. On the other hand, these by-products are rich in many nutrient components, including proteins, sugars, oligosaccharides, minerals, and soy isoflavones; therefore, they have value-added potential.

In recent years, oligosaccharides, as an excellent microbiota-accessible carbohydrate, have exhibited important prebiotic properties via their fermentation in the intestine, which is attributed to them acting as carbon sources for specific probiotics, promoting the production of SCFAs, and regulating the gut microbiota [5]. Soybean oligosaccharides (SBOS) isolated from soybean have been widely reported, which consist mainly of raffinose, stachyose, and sucrose [6]. Many studies have indicated that SBOS could significantly reduce abnormal blood sugar, lipid levels, and oxidative stress. In addition, they could competitively inhibit potential pathogenic bacteria, improve insulin resistance, and enhance the immune system to prevent various diseases [7,8,9,10,11,12,13]. Recently, tofu wastewater (also known as yellow water due to its yellow color), one of the main by-products of tofu production, has been reported to contain SBOS [14]. Various technologies, such as reverse osmosis and nanofiltration membranes, have been used to separate SBOS from tofu wastewater [11]. However, the exact molecular structures of SBOS from tofu wastewater have rarely been reported [1]. In addition, the fermentability of tofu wastewater SBOS on human gut microbes, either as mixtures or single fractions, has yet to be reported [6,12].

In the present study, crude SBOS were isolated from wastewater of tofu production and subsequently separated into three sub-fractions, Z1, Z2, and Z3, using Sephadex G-15 column chromatography. The structural features of each fraction were confirmed using corresponding standards. The impacts of SBOS and its sub-fractions on human intestinal microbiota were also investigated. The comprehensive analysis of the generated SCFAs and gut microbiota compositions was conducted using gas chromatography (GC) and high-throughput 16S rDNA gene sequencing. This study aimed to enhance the value-added applications of soybean wastewater and provide a reference for the exploitation and utilization of other soybean by-products in the food industry.

## 2. Materials and Methods

### 2.1. Materials and Chemical Reagents

**Figure 1:**
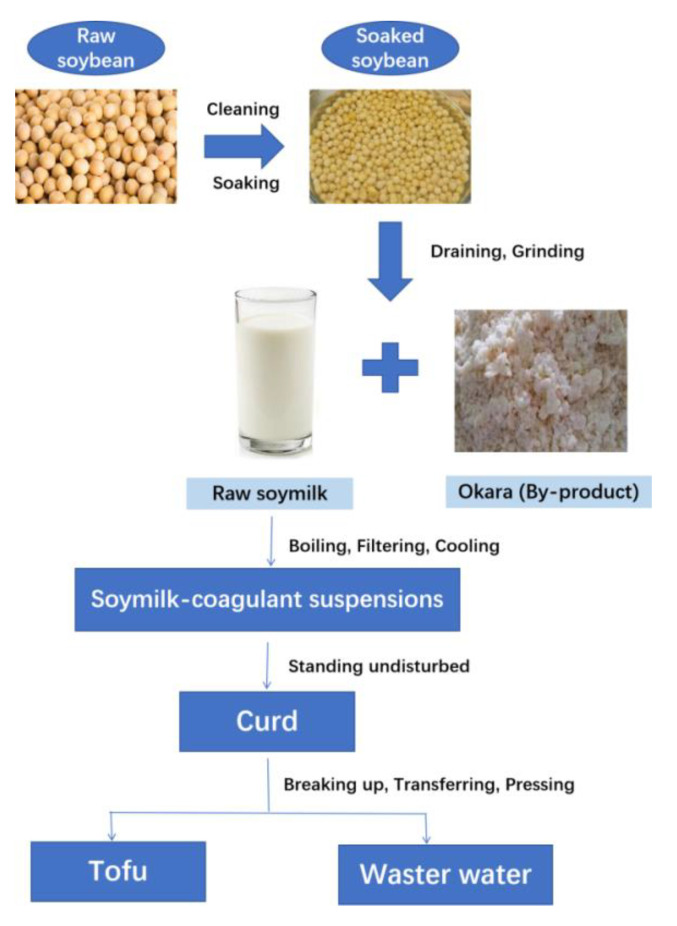
Schematic flow chart of the production of tofu from soybeans.

Wastewater of tofu production was provided by Tianjin Shanhaiguan Bean Products Co., LTD (Tianjin, China). The flow chart of tofu production is demonstrated in Figure 1. Moreover, 2-ethylbutyric acid and volatile free acid standard mix including acetic acid, propionic acid, isobutyric acid, butyric acid, isovaleric acid, valeric acid, isocaproic acid, caproic acid, and heptanoic acid were purchased from Sigma-Aldrich Chemical Co. (Shanghai, China). All the other chemicals and solvents used in the study were of analytical grade.

### 2.2. Isolation and Fractionation of SBOS

Yellow wastewater from tofu production was condensed (1/5 of its original volume) and precipitated using three times the volume of cold ethanol (4 °C). The supernatant was then freeze-dried and termed crude SBOS. The crude SBOS isolated from wastewater of tofu production were redissolved in distilled water (30 mg/mL), and 2 mL solution was passed through the Sephadex G-15 column (1.0 × 70 cm, Sigma, Shanghai, China) at a flow rate of 0.5 mL/min with ultra-pure water. The SBOS were pooled based on the elution curve monitored by the phenol–sulfuric acid method [15].

### 2.3. Oligosaccharide Profile Analysis

Each tube solution containing SBOS obtained from the Sephadex G-15 column was determined using a Shimadzu HPLC system (Prominence LC-20A, Kyoto, Japan) equipped with an Ultrahydrogel TM DP guard column, Ultrahydrogel TM 250 column (7.8 × 300 mm, Waters, Milford, MA, USA), and Ultrahydrogel TM DP 120A column (7.8 × 300 mm, Waters, Milford, MA, USA) in series, coupled with a refractive index detector (RI). The running conditions were as follows: 20 μL oligosaccharide solution (3 mg/mL), detecting temperature at 40 °C, flow rate of 0.5 mL/min, and Milli-Q water as the mobile phase. According to the results, the different sub-fractions collected were designated Z1, Z2, and Z3 in the order of increased retention volume.

### 2.4. In Vitro Fermentation of SBOS

#### 2.4.1. Preparation of Human Intestinal Microbiota and Medium

This study used a high concentration of fecal inoculum from human fecal samples to provide the microbiota and as the main source of nutrients for the bacteria [16]. The fresh human feces were collected from three healthy volunteers aged 20–28 who had not been treated with antibiotics in the last three months. Fecal samples were collected as described by Young-Do N et al. [17]. Approximately 5 g of stool sample was collected into sterile plastic containers by the participants themselves and immediately brought to the experimental laboratory. The feces were mixed well in an equal amount in an anaerobic incubator and stored at –80 °C until further processing.

The protocol followed Ding et al. [18], with minor modifications. In brief, 1 L anaerobic incubation medium (AIM) containing 50 mg CaCl_2_, 2 mg CoCl·6H_2_O, 20 mg FeSO_4_, 900 mg K_2_HPO_4_, 50 mg MgSO_4_, 20 mg MnSO_4_·H_2_O, 900 mg NaCl, 4000 mg Na_2_CO_3_, 900 mg (NH_4_)_2_SO_4_, 20 mg MnSO_4_·H_2_O, and 1 mg resazurin was sterilized at 121 °C for 20 min (pH 6.8) after boiling and flushing with mixed gas (10% CO_2_, 10% H_2_, and 80% N_2_). Additionally, vitamins including 0.05 mg biotin, 2 mg calcium D-pantothenate, 0.005 mg cobalamine, 0.05 mg folic acid, 2 mg nicotinamide, 0.1 mg para-aminobenzoic acid, 2 mg vitamin B2, 2 mg vitamin B6, 2 mg vitamin B1, and 1000 mg L-Cysteine HCl solutions were sterilized using 0.22 μm filters, and then mixed with the autoclaved medium in the anaerobic chamber (Shanghai CIMO Medical Instrument Manufacturing Co., Ltd., Shanghai, China).

#### 2.4.2. In Vitro Fermentation

In the fermentation system, oligosaccharides including SBOS, raffinose, stachyose, sucrose, C1 (a mass ratio of raffinose and stachyose of 1:3), and C2 (a mass ratio of raffinose, stachyose and sucrose of 1:3:4), which was based on a previous study [19], were the sole carbon source, with a concentration of 1% (*w*/*v*), and 10% (*w*/*v*) of feces (fecal mixture from three volunteers) was selected for inoculation. Specifically, 0.04 g oligosaccharide samples were thoroughly dissolved in 4 mL AIM, and then mixed with feces (0.4 g) in an anaerobic chamber. Subsequently, the mixture was introduced in different anaerobic sealed bags for 48 h at 37 °C in a thermostat incubator (ZWY-103B, Shanghai Zhicheng Analytical Instrument Manufacturing Co., Ltd., Shanghai, China). The culture fluid was collected at 0 h, 12 h, 24 h, and 48 h, respectively, and centrifuged at 11,000 r/min for 20 min. The supernatant was used to determine SCFAs and changes in molecular weight, while the precipitate was used to investigate the changes in human gut microbiota. Fructo-oligosaccharides (FOS) served as the positive control, while the negative control (NC) only contained feces in AIM without carbon sources added.

### 2.5. Determination of SCFAs

The supernatant of the fermentation culture was filtrated through a 0.22 μm membrane, and the SCFAs were analyzed by a GC (Bruker 450GC, Billerica, MA, USA) system equipped with a Nukol TM Fused Silica Capillary Column (60 m × 0.25 mm × 0.25 μm) and flame ionization detector (FID). The temperatures of the injector and detector were 200 °C and 250 °C, respectively. The standard curves were established with volatile free fatty acid mixed standard, and 2-ethylbutyric acid was added as an internal standard.

### 2.6. Changes in Mw of SBOS

The supernatant of the fermentation culture was filtrated through a 0.22 μm membrane and the changes in the oligosaccharide profiles of SBOS and sub-fractions under the different fermentation times were tracked by HPLC. The analysis method was the same as described in Section 2.3.

### 2.7. 16S rDNA Gene Sequencing of Gut Microbiota

The genomic DNA was extracted according to the method of Chen et al. [20]. The PCR amplification was performed on the V3-V4 hypervariable regions of the bacterial 16S rDNA genes, which used the forward primer 341F (5′-CCTAYGGGRBGCASCAG-3′) and the reverse primer 806R (5′-GGACTACNNGGGTATCTAAT-3′). PCR products were accumulated using the QIAquick PCR Purification Kit (QIAGEN, Hilden, Germany). The 16S rDNA amplicon sequencing was performed on the Illumina NovaSeq PE250 platform (Novogene Bioinformatics Technology Co., Ltd., Beijing, China). The Ion Plus Fragment Library Kit 48 rxns library construction kit of Thermofisher Company (Waltham, MA, USA) was used to construct the library. After the library was qualified by Qubit quantification and a library test, the Ion S5TMXL of Thermofisher was used for computer sequencing. Clean reads of all samples were clustered by Uparse software (Uparse v7.0.1001, Robert C Edgar, Tiburon, CA, USA,) at a 97.0% similarity level to obtain OTUs. Alpha diversity analysis was performed using ACE, Chao 1, rarefaction curves, and Shannon indexes. The weighted unifrac algorithm was used for the analysis of PCoA. The analysis of LefSe was performed with a threshold >4.0.

### 2.8. Statistical Analysis

The data were expressed as means ± standard deviations (STD) based on triplicate tests. The statistical significance of differences (*p* < 0.05) was analyzed by ANOVA using SPSS 25.0 software (IBM Corp, Chicago, IL, USA).

## 3. Results

### 3.1. Isolation and Fractionation

**Figure 2:**
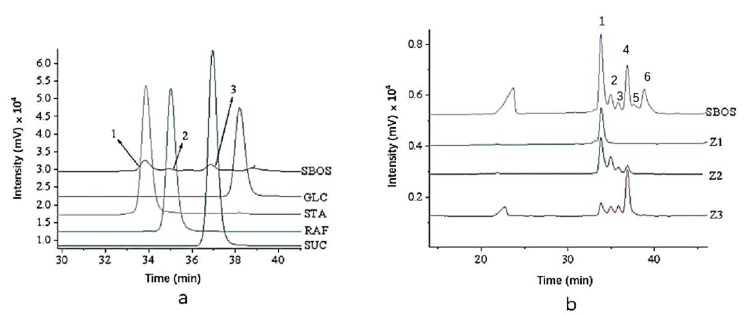
The HPLC chromatogram of SBOS, glucose, sucrose, raffinose, and stachyose (**a**); the HPLC chromatogram of three sub-fractions Z1, Z2, and Z3 (**b**).

As shown in Figure 2b, crude SBOS contained six peaks. The percentages of peaks 1 to 6 were 40.1%, 9.2%, 4.8%, 23.4%, 5.0%, and 17.5%, respectively, according to the ratios of peak areas. Compared with standards, peak 1, peak 2, and peak 3 (Figure 2a) in SBOS corresponded to stachyose, raffinose, and sucrose, respectively. The SBOS were then further separated and purified by the Sephadex G-15 column, from which three components were obtained, named Z1, Z2, and Z3, with yields of 14%, 13%, and 17%, respectively, as shown in Figure 2b. The contents of stachyose, raffinose, sucrose, and glucose were determined based on the peak area ratios of each component in Z1, Z2, and Z3. As shown in Figure 2b and Table 1, Z1 was stachyose (100%); Z2 contained stachyose (60.41%), raffinose (28.07%), and sucrose (11.52%); Z3 was composed of sucrose (67.69%), stachyose (19.25%), and raffinose (13.06%).

### 3.2. In Vitro Fermentation

#### 3.2.1. SCFAs Production

**Figure 3:**
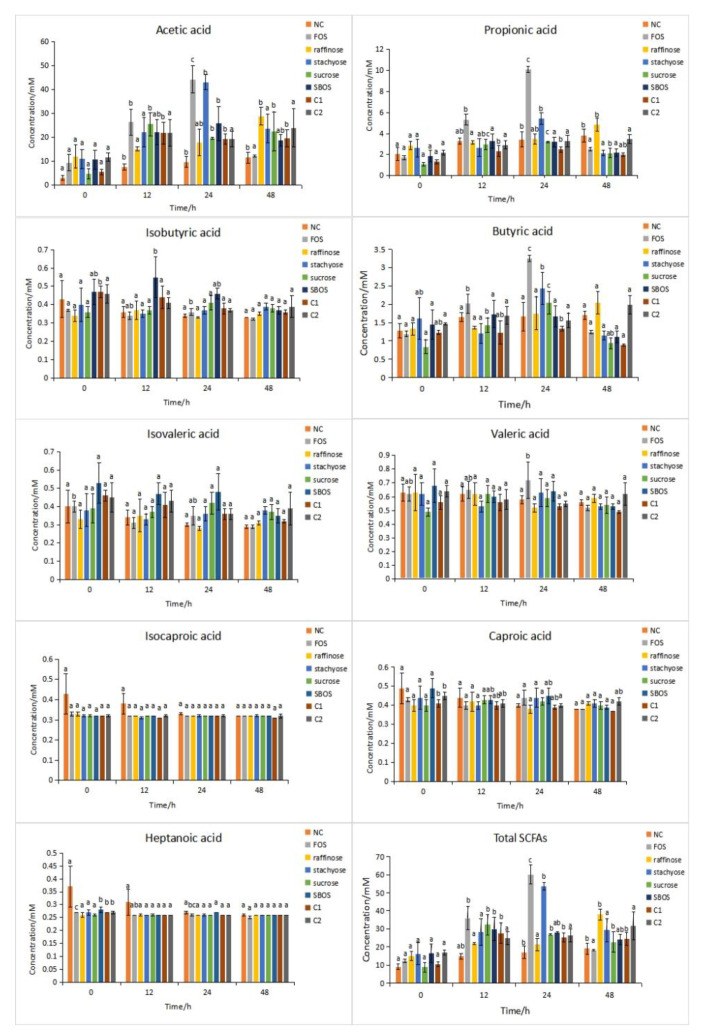
Concentrations (mM) of SCFAs during in vitro fermentation at different time points. One-way ANOVA and Duncan’s test were used to evaluate significant differences for SCFAs production. Different lowercase letters with different times were significantly different (*p* < 0.05).

According to the standard curves of SCFAs in mixed acid standards, as shown in Table 2, the concentrations of SCFAs including acetic acid, propionic acid, isobutyric acid, butyric acid, isovaleric acid, valeric acid, isocaproic acid, caproic acid, and heptanoic acid of FOS, raffinose, stachyose, sucrose, SBOS, C1, and C2 generated during in vitro fermentation at different time points are presented in Figure 3.

Acetic acid, propionic acid, and butyric acid were the main SCFAs obtained by the in vitro fermentation of oligosaccharides. Compared with the negative control (NC), the acetic acid, propionic acid, and butyric acid production from FOS were increased significantly (*p* < 0.05) at 24 h. The same trends were observed in stachyose, but the concentration of SCFAs was slightly lower than FOS. The acetic acid content in the sucrose group reached the maximum at 12 h fermentation, which was 25.50 ± 4.90 mM, slightly lower than that in the FOS group (26.33 ± 5.42 mM). The content of acetic acid produced by the SBOS group was increased to 25.75 ± 7.24 mM at 24 h fermentation, which reached a maximum. At 24 h fermentation, the amounts of propionic acid produced by FOS, stachyose, and SBOS were 10.07 ± 0.29 mM, 5.42 ± 0.53 mM, 3.19 ± 0.44 mM, respectively. The content of butyric acid produced by SBOS at 12 h reached the highest at 1.73 ± 0.37 mM, which was higher than the stachyose and raffinose groups but lower than the FOS group.

Previous studies showed that the major SCFAs of soybean meal oligosaccharides (SMO) were acetic, propionic, and butyric acids, which accounted for approximately 94.4% of the total SCFAs [21]. The total SCFAs produced by stachyose and raffinose were higher than SMO. SCFAs play several roles in animal and human metabolism. Among them, acetic acid is the main SCFA in the large intestine, which is absorbed by the colonic epithelium cells and then transported to the heart, brain, and nerve tissues for energy [22]. Butyric acid is the primary source of energy for colon epithelial cells, which maintains the integrity of the intestinal mucosal barrier. It has the unique ability to promote the normal phenotype of colon cells by repairing damaged DNA [23]. Propionic acid can inhibit the synthesis of cholesterol [24]. Moreover, as early as 1991, a study showed that the oligosaccharides fructans, stachyose, lactose, and raffinose could pass into the large intestine and were readily fermented to produce SCFAs [25].

#### 3.2.2. Changes in Mw during Fermentation

**Figure 4:**
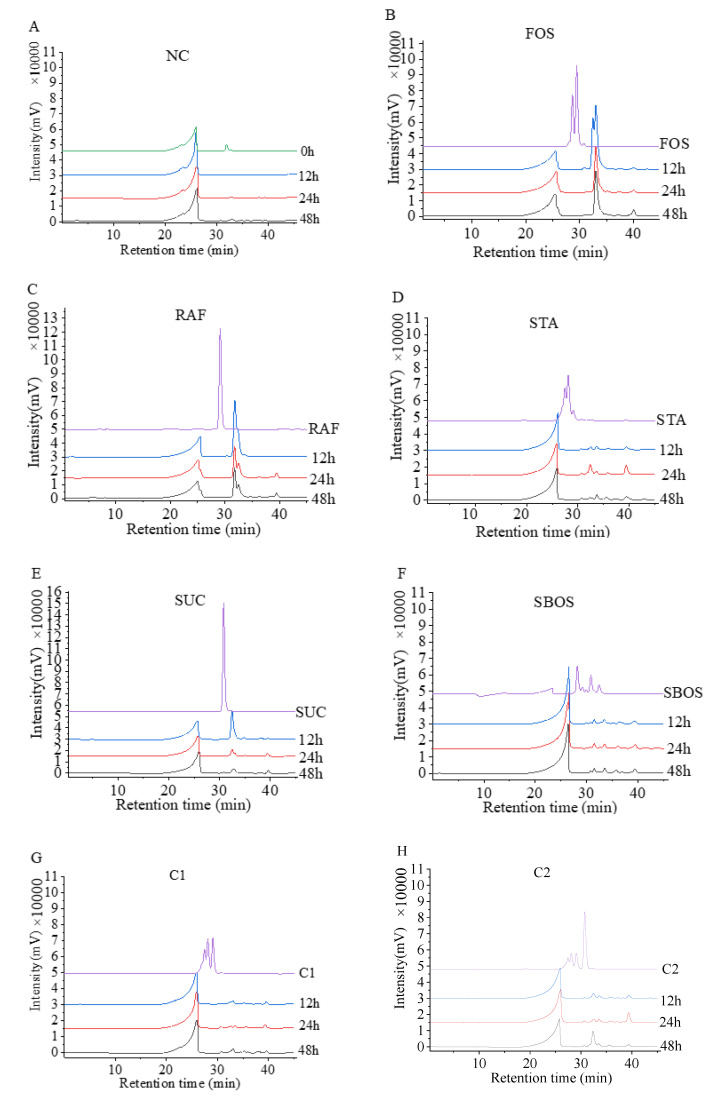
Mw distribution of NC (**A**) and oligosaccharides FOS (**B**), raffinose (RAF) (**C**), stachyose (STA) (**D**), sucrose (SUC) (**E**), SBOS (**F**), C1 (**G**), C2 (**H**) before and after simulated intestinal fermentation by human fecal microbiota.

The Mw changes of the NC group and different oligosaccharides during the fermentation are shown in Figure 4. With the increase in fermentation time, the Mw of oligosaccharides decreased significantly. As demonstrated in Figure 4, the peak time of oligosaccharides changed and the peak area was decreased during the fermentation, indicating that they could be utilized and degraded by the colonic microbiota. As shown in Figure 4B,C,E, the fermentation ability of FOS and stachyose was weaker than that of sucrose. Interestingly, there were no large peaks after 12 h fermentation for stachyose, SBOS, C1, or C2 groups, which indicated that these oligosaccharides could be quickly utilized by colonic microorganisms and most of them were degraded. In general, the ability of intestinal microorganisms to use oligosaccharides for fermentation was affected by the types, molecular weights, and the proportions of each component of oligosaccharides. In the present study, SBOS could be quickly utilized and degraded by colonic microorganisms. Similarly, recent studies observed that oligosaccharides were rapidly degraded during in vitro fermentation [26], which is consistent with our results.

#### 3.2.3. Changes in Microbiota Composition

Dietary carbohydrates can be selectively utilized by gut microbiota to modulate the composition of the gut microbiota and activate the proliferation of probiotics associated with host health [27]. In the present study, the effects of FOS, raffinose, stachyose, and SBOS on the gut microbiota were examined using the sequences of the 16S rDNA gene (repeated three times).

**Figure 5:**
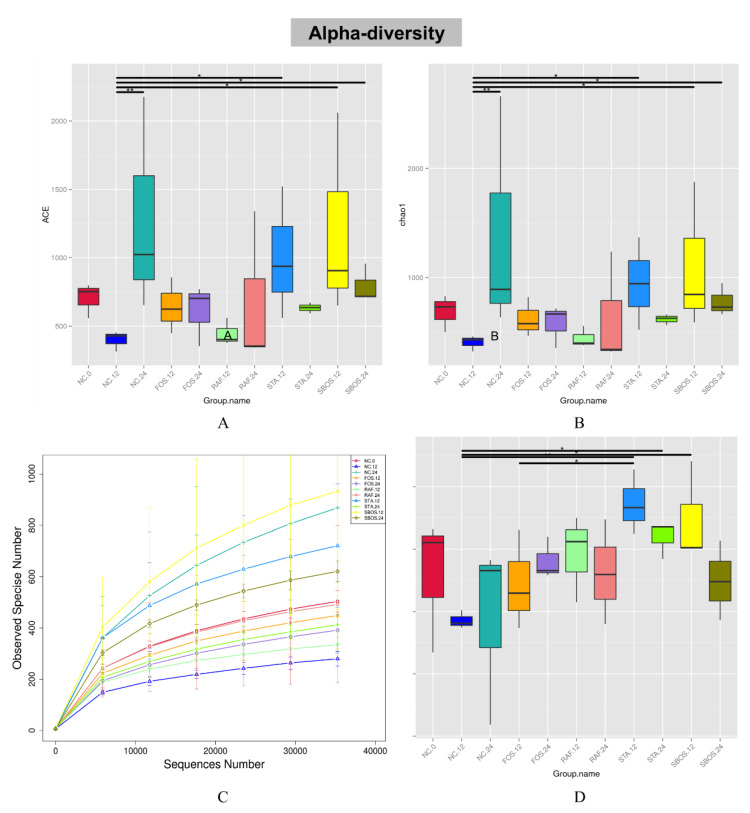
Alpha diversity including ACE (**A**), Chao 1 (**B**), rarefaction curves (**C**), and Shannon index (**D**).

The alpha diversity of the gut microbiota can reflect microbial species diversity, including richness and uniformity. The rarefaction curves, which were used to confirm whether the sample sizes and sequencing depth were adequate to study the intestinal microbiota, are shown in Figure 5C. The results showed that with the increase in sequencing depth and sample size, no additional OTUs were detected, revealing that the data of the gut microbial community were sufficient and credible to meet the requirements for data analysis. Here, the ACE, Chao 1, and Shannon indexes were used to evaluate the complexity of the microbial communities. As shown in Figure 5A,B, compared with the NC.12 group, the ACE and Chao1 indexes were significantly increased in the STA.12, SBOS.12, and SBOS.24 groups (*p* < 0.05), indicating that the treatments of these groups could increase the community richness of the fecal microbiota. In addition, compared with the NC.12 group, the Shannon index was significantly increased in the STA.12, STA.24, and SBOS.12 groups, suggesting that the microbial community diversity was raised with the treatments of these groups.

**Figure 6:**
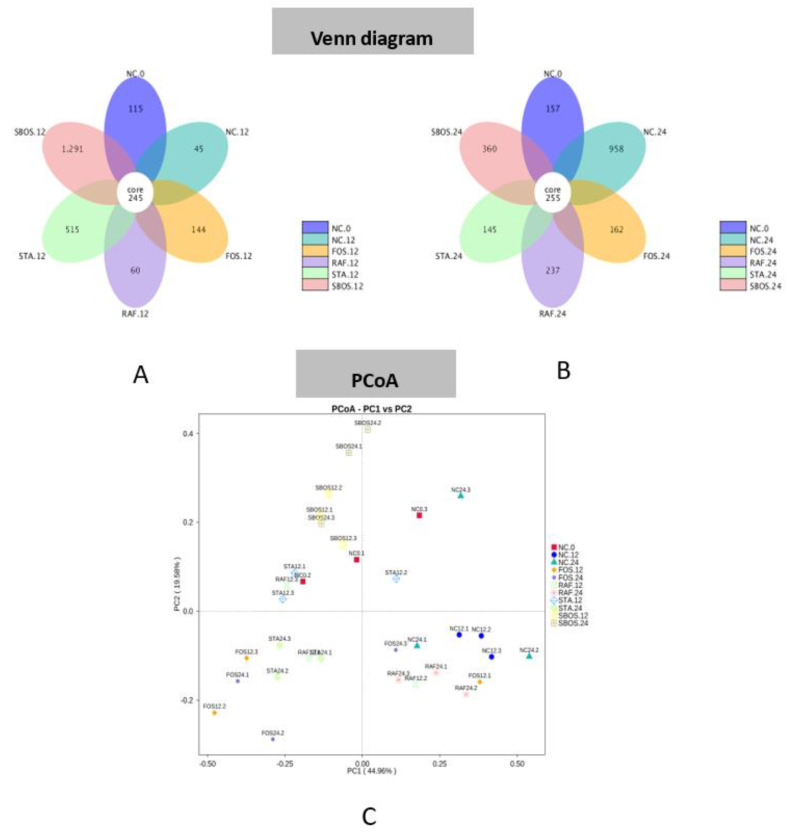
Venn diagram of OTUs at 12 (**A**) and 24 h (**B**) of fermentation and PCoA analysis (**C**) of gut microbiota based on weighted unifrac distance.

A Venn diagram is presented in Figure 6A,B that shows overlap in the observed OTUs among these samples. There were 245 shared OTUs at 12 h and 255 shared OTUs at 24 h of fermentation. A higher number of OTUs were retained at 24 h than 12 h of fermentation. PCoA was carried out based on the weighted unifrac distances to analyze the taxonomy in the oligosaccharide and NC groups, as shown in Figure 6C. The results suggested that the clusters of the oligosaccharide groups intertwined mutually but detached from those of the NC.12 group, which is consistent with the results of alpha diversity.

**Figure 7:**
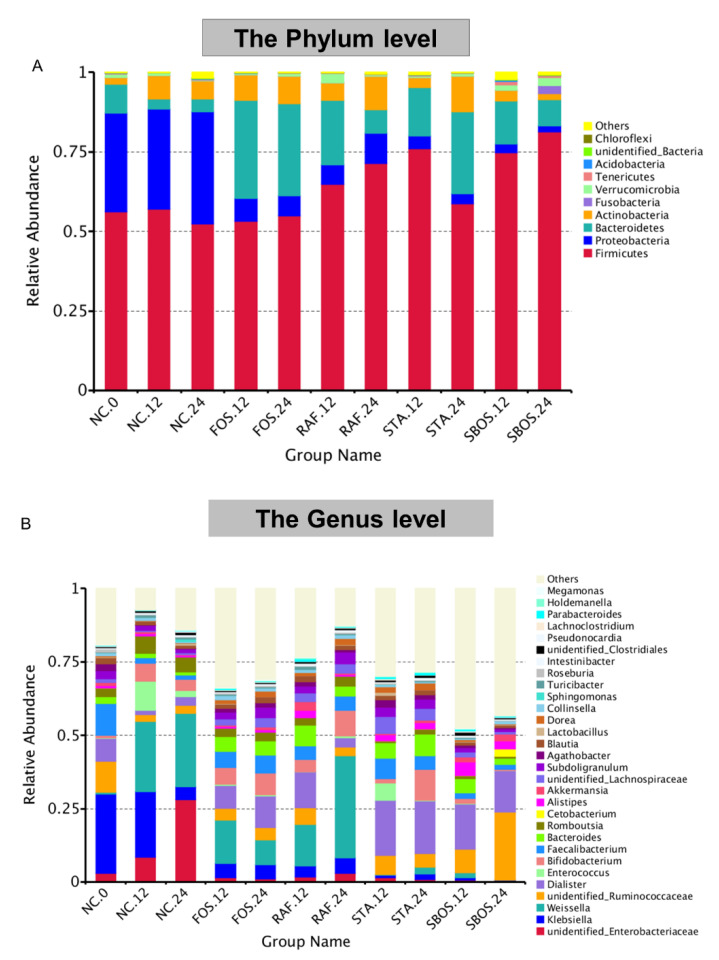
Intestinal microbial community structure: bar plot at the phylum level (**A**) and the genus level (**B**).

In Figure 7, the gut microbiota composition structures at the phylum and genus levels for all the groups are displayed. The microbiota community was mainly composed of Firmicutes, Bacteroidetes, Actinobacteria, and Proteobacteria at the phylum level (Figure 7A). After 24 h, the relative abundances of Bacteroidetes and Firmicutes decreased, while those of Actinobacteria and Proteobacteria increased in the control group. Compared with the NC.0 group, the relative abundance of Firmicutes was significantly increased in the SBOS group, while the abundance of Proteobacteria was reduced, indicating that SBOS could promote the regulation of the intestinal microenvironment, which is consistent with the results of Arseneau et al. [28]. Studies have confirmed that a bloom of Proteobacteria is a manifestation of dysbiosis or instability of the gut microbial community [29]. In the FOS.12 and FOS.24 groups, the relative abundance of Proteobacteria was slightly reduced, while that of Bacteroidetes and Actinobacteria was significantly increased.

To further identify the specific genus of interest to metabolize SBOS, the abundance of the genera was evaluated, as shown in Figure 7B. At the genus level, the gut bacteria before fermentation were mainly composed of *Klebsiella*, *unidentified_Ruminococcaceae*, *Dialister*, *Faecalibacterium*, *Bacteroides*, *Roseburia*, *Agathobacter*, and so on. After 24 h fermentation, six genera, namely *unidentified_Enterobacteriaceae*, *Weissella*, *Enterococcus*, *Bifidobacterium*, *Roseburia*, and *Sphingomonas*, were obviously increased, whereas *Klebsiella*, *Faecalibacterium*, and *Dialister* were decreased compared to the control group. Compared with the NC.12 group, STA.12 and SBOS.12 decreased the relative abundance of genera *Klebsiella*, *Weissella*, *Enterococcus*, and *Romboutsia*, and promoted the proliferation of *Alistipes*, *Dialister*, *Faecalibacteerium*, and *Bacteroides*. In addition, SBOS also significantly increased the abundance of *Akkermansia*, which could improve the inflammatory response and protect intestinal epithelial cells [30]. Moreover, the genera Dialister, Faecalibacterium, *Bacteroides*, and *Bifidobacterium* were increased in the FOS.24, RAF.24, and STA.24 groups compared to the NC.24 group. Among them, *Bifidobacteria* is the most often utilized prebiotic oligosaccharide, which can beneficially affect host health [30,31]. One study has shown that feeding SBOS resulted in significantly higher *Bifidobacteria* and *Clostridium perfringens* than in the control group [32].

**Figure 8:**
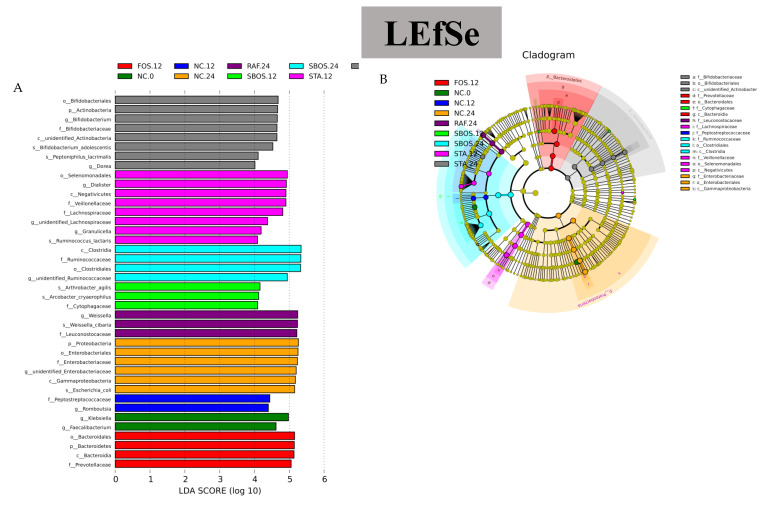
Distribution histogram (**A**) based on linear discriminant analysis (LDA): LDA score threshold >4.0 and cladogram (**B**) representation of taxa using linear discriminant analysis effect size (LEfSe).

The distribution histogram based on LDA scores is shown in Figure 8A, which estimates the intestinal microbiota diversity and reveals the dominant microorganisms in the oligosaccharide and NC groups. These results indicate that *Klebsiella*, *Faecalibacterium* in the NC 0 group and *Peptostreptococcaceae*, *Romboutsia* in the NC.12 group had a significant influence. Meanwhile, in the NC.24 group, *Proteobacteria*, *Enterobacteriales*, *Enterobacteriaceae*, *Gammaproteobacteria*, and *Escherichia-coli* had an important influence in the dominant community. However, many beneficial bacteria were found after oligosaccharide treatment. Among them, *Bacteroidales*, *Bacteroidetes*, *Bacteroidia*, and *Prevotellaceae* had an important influence in the FOS.12 group. *Prevotellaceae* has an important role in the hydrolysis of dietary fiber among intestinal microorganisms, and its activity can be significantly increased by carbohydrate treatment [33]. *Bacteroidetes* have a significant role in promoting metabolism and immune function in obese people [34]. In the RAF.24 group, there was one dominant family and two types of dominant genera: *Leuconostocaceae, Weissella*, and *Weissella-cibaria*. There were eight types of dominant genera for the stachyose groups after 12 h and 24 h, respectively, including *Granulicella*, *Dorea*, *Bifidobacterium*, *Ruminococcus*, etc. In the SBOS groups, *Clostridia*, *Ruminococcaceae*, *Clostridiales*, *Arthrobacter*, *Cytophagaceae*, etc., had an important influence. SCFAs are mainly produced by microbial fermentation and are the main products of intestinal bacterial metabolism. *Akkermansia, Ruminococcus*, and *Prevotella* have been reported to be associated with the degradation of carbohydrates and the production of SCFAs [35,36]. In addition, the results of the evolutionary branch graph of LEfSe are presented in Figure 8B. In the NC group, the branches of Proteobacteria were the major microbiota. In comparison, the predominant gut microbiota, such as Firmicutes and Bcteroidetes, played a critical role in fermentation in the oligosaccharide group. These results were in accordance with the analysis of the microbiota composition shifts at the phylum and genus levels. Therefore, SBOS could be used as a dietary supplement to improve gut microbiota and promote gut health.

### 3.3. Correlation of Structure and Prebiotic Properties

According to a previous study, the utilization rate of oligosaccharides may depend on their structures, including the degree of polymerization, degree of glycoside binding, and branching [37]. For instance, the prebiotic activity of pectin oligosaccharides (POSs) was positively correlated with the branching degree (galactose: rhamnose and arabinose: rhamnose molar ratios) and the content of neutral sugars, especially galactose and arabinose [38]. Moreover, many recent studies indicated that low-Mw or de-polymerized oligosaccharides exhibit better colonic persistence and thus have increased fermentability by intestinal microbial communities [39]. In addition, Wu et al. summarized the structural features, interactions with the gut microbiota, and anti-tumor activity of eight common natural oligosaccharides, namely FOS, human milk oligosaccharides (HMOS), galactooligosaccharides (GOS), xylooligosaccharides (XOS), chitinoligosaccharides (NACOS), arabinoxylo-oligosaccharides (AXOS), mannan-oligosaccharides (MOS), and isomaltooligosaccharides (IMOS), with low-viscosity oligosaccharides, which displayed a fast fermentation rate by gut microbiota [40].

## 4. Conclusions

In conclusion, SBOS could be extracted from tofu wastewater and purified into three sub-fractions, named Z1, Z2, and Z3. Among them, Z1 was identified as stachyose, while Z2 and Z3 contained stachyose, raffinose, and sucrose with different amounts. Thus, SBOS were confirmed to be an oligosaccharide mixture including tetrasaccharide, trisaccharide, and disaccharides. In addition, the results of in vitro fermentation showed that SBOS could be degraded and significantly promoted the production of SCFAs, especially acetic, propionic, and butyric acids. Additionally, SBOS could modulate the intestinal microbiota composition and significantly increase the relative abundance of beneficial bacteria. These results provide a good understanding of the effects of SBOS on the fermentability of the human gut and SBOS can be developed as potential functional ingredients to maintain human intestinal health. Although we can confirm, to a certain extent, that SBOS are a possible prebiotic capable of promoting intestinal health, the in vitro fermentation model also has some limitations—for instance, microorganisms are not easily controlled during fermentation; intestinal absorption, digestive secretion, and the intestinal defense system are not considered. Therefore, in vivo experiments on SBOS should be conducted to further verify their probiotic potential.

## Figures and Tables

**Table 1:** Peak area ratios of stachyose, raffinose, sucrose in Z1–Z3 components and monosaccharide composition.

Fractions	Yield (%)	Peak Area Ratios (%)		
		Stachyose ^1^	Raffinose ^2^	Sucrose ^3^
Z1	14	100.00	-	-
Z2	13	60.41	28.07	11.52
Z3	17	19.25	13.06	67.69

^1^ Rt: 33.87 min; ^2^ Rt: 35.03 min; ^3^ Rt: 36.91 min.

**Table 2:** Standard curves of SCFAs in mixed acid standards.

SCFAs	Standard Curve	R^2^
Acetic acid	y = 0.1556x − 0.0057	0.9916
Propionic acid	y = 0.3400x − 0.0216	0.9905
Isobutyric acid	y = 0.4474x − 0.0257	0.9947
Butyric acid	y = 0.4396x − 0.0282	0.9924
Isovaleric acid	y = 0.4801x − 0.0259	0.9958
Valeric acid	y = 0.4696x − 0.0413	0.9921
Isocaproic acid	y = 0.4630x − 0.0359	0.9959
Caproic acid	y = 0.4582x − 0.0359	0.9953
Heptanoic acid	y = 0.4388x − 0.0310	0.9950

## Data Availability

No new data were created or analyzed in this study. Data sharing is not applicable to this article.
